# Supplementary material for: Identification of photoperiod-induced specific miRNAs in the adrenal glands of Sunite sheep (Ovis aries)
Source: Front Vet Sci. 2022 Jul 22;9:888207. doi: 10.3389/fvets.2022.888207 (PMC9354845; doi:10.3389/fvets.2022.888207)
Supplement: Supplementary file 1 [file Data_Sheet_1.ZIP › Supplementary file/miRDeep2 file.zip!/miRDeep2 file/Novel_Structure/LP42A1/Novel_560.pdf]

Provisional ID	:	NC_019464.2_14809
Score total	:	406.5
Score for star read(s)	:	3.9
Score for read counts	:	398.8
Score for mfe	:	2.2
Score for randfold	:	1.6
Score for cons. seed	:	
Total read count	:	794
Mature read count	:	793
Loop read count	:	0
Star read count	:	1

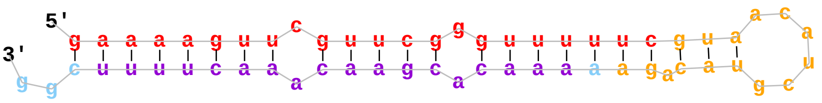

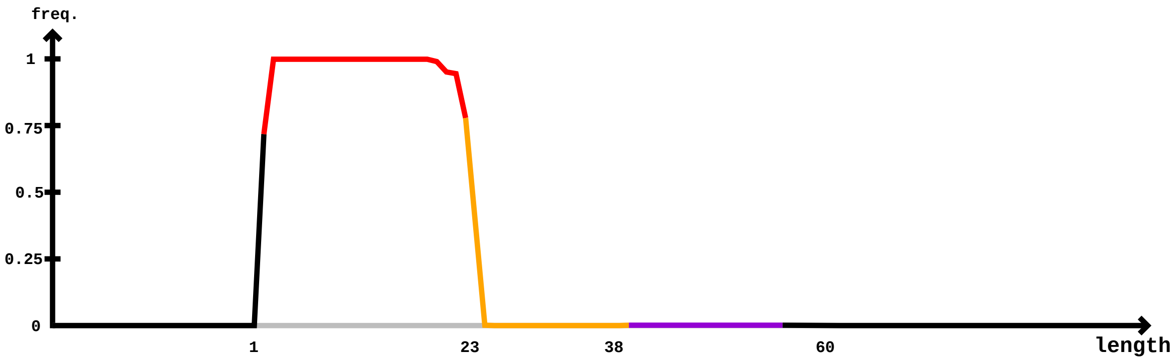

Mature Star

```
5'- auuaucauauuggguuggcugaaaaguucguucggguuuuucguaacaucguacagaaaaacacgaacaaacuuuucggccaacccaguauuucauucaucugcguuuuccu   -3'   obs
    auuaucauauuggguuggcugaaaaguucguucggguuuuucguaacaucguacagaaaaacacgaacaaacuuuucggccaacccaguauuucauucaucugcguuuuccu         exp
    ......((((((((((((((((((((((.(((((.((((((((((......))).))))))).))))).)))))))))))))))))))))).....................    reads   mm   sample
    ....................gaaaaguucguucggguu..........................................................................    7       0    seq
    ....................gaaaaguucguucggguuu.........................................................................    31      0    seq
    ....................gaaaaguucguuUggguuuuu.......................................................................    9       1    seq
    ....................gaaaaguucguuUggguuuuuc......................................................................    1       1    seq
    ....................gaaaaguucguucggguuuuuc......................................................................    521     0    seq
    ....................Aaaaaguucguucggguuuuuc......................................................................    1       1    seq
    .....................aaaaguucguucggguuuu........................................................................    5       0    seq
    .....................aaaaguucguucggguuuuC.......................................................................    15      1    seq
    .....................aaaaguucguucggguuuuu.......................................................................    108     0    seq
    .....................aaaaguucguucggguuuuuc......................................................................    84      0    seq
    .....................aaaaguucguucggguuuuCc......................................................................    1       1    seq
    .....................aaaaguucguuUggguuuuucg.....................................................................    3       1    seq
    .....................aaaaguucguucggguuuuucU.....................................................................    6       1    seq
    .....................aaaaguucguuUggguuuuucgu....................................................................    1       1    seq
    ..........................................................aaacacgaacaaacuuuu....................................    1       0    seq
```
